# Gastric sleeve and gastric bypass: changes in weight after two-year follow-up - which is more effective?

**DOI:** 10.1590/0102-67202025000045e1914

**Published:** 2025-12-08

**Authors:** Alexandra Rabello FREIRE, Flávio KREIMER, Denise Sandrelly Cavalcanti de LIMA, Cinthia Katiane Martins CALADO, Silvia Alves da SILVA, Maria Goretti Pessoa de Araújo BURGOS

**Affiliations:** 1 Universidade Federal de Pernambuco, Clinical Nutrition Unit, University Hospital - Recife (PE), Brazil.; 2 Universidade Federal de Pernambuco, Department of Surgery - Recife (PE), Brazil.; 3 Universidade Federal de Pernambuco, Vitoria Academic Center - Vitória de Santo Antão (PE), Brazil.; 4 Universidade Federal de Pernambuco, Nutrition Department - Recife (PE), Brazil.

**Keywords:** Bariatric Surgery, Obesity, Weight Loss, Weight Gain, Cirurgia Bariátrica, Obesidade, Redução de Peso, Aumento de Peso

## Abstract

**Background:**

Bariatric surgery is currently the gold standard for the treatment of obesity. However, weight recurrence varies among the different surgical methods.

**Aims:**

To compare changes in weight one and two years after bariatric surgery considering the gastric bypass and gastric sleeve methods.

**Methods:**

A cross-sectional study was conducted at a hospital with adults of both sexes followed up for two years after surgery. Anthropometric, sociodemographic, clinical, and lifestyle characteristics were analyzed.

**Results:**

A total of 184 patients, predominantly women (82.1%), were assessed (136 submitted to gastric sleeve and 48 to gastric bypass). Good adherence to the multivitamin, but not to diet or physical activity, was verified in both groups. The percentages of weight loss and excess weight loss were higher in the gastric bypass group (one year after surgery: p<0.001 and p=0.010, respectively; two years after surgery: p<0.001 and p<0.001, respectively). Average weight gain was 2.37 kg and higher after gastric sleeve (p=0.042), whereas no difference between methods was found for the percentage of weight recurrence. Weight loss and recurrence at the two-year follow-up were influenced by diet in both groups. The percentage of weight loss was higher after gastric bypass one and two years after surgery. Weight recurrence was higher after the gastric sleeve method, without interfering with the surgical success of the technique.

**Conclusions:**

We verified greater efficacy in the gastric bypass technique in terms of weight loss at 12 and 24 months postoperatively. Weight recurrence was found 24 months after both methods, especially in the gastric sleeve group, without constituting surgical failure.

## ARTICLE HIGHLIGHTS

• Gastric bypass promoted greater weight loss at 12 and 24 months compared to the gastric sleeve method;

• Weight recurrence two years after surgery was greater in the gastric sleeve group;

• Weight recurrence was found with both surgical techniques, but without constituting surgical failure;

• Adherence to diet and physical activity exerted an influence on both weight loss and recurrence.

## Summary

**CENTRAL MESSAGE:**

Bariatric surgery is considered the gold standard for the treatment of obesity that is difficult to control clinically. Such surgery is recommended by the main guidelines due to its short- and long-term effectiveness. Among the authorized surgical techniques, gastric bypass and gastric sleeve are the most widely performed throughout the world, with an increase in the indication for the latter. The results of these surgical interventions are satisfactory, with significant reductions in excess body weight. Weight recurrence is mainly associated with unhealthy lifestyle and/or long-term physiological and anatomical adaptations. Thus, studies comparing weight recurrence between the two surgical techniques are important so that the indication of each technique occurs in an individualized and assertive manner.

**PERSPECTIVES:**

The type of surgical technique exerted an influence on the percentage of weight loss (%WL) and percentage of excess weight loss (%EWL) at 12 and 24 months. Gastric bypass was better for achieving the surgical objective in both periods. Weight recurrence was only found at 24 months in both groups and was greater in the gastric sleeve group, but without constituting surgical failure. These results are relevant, and studies analyzing long-term weight recurrence are needed.

## VISUAL ABSTRACT

**Figure:**
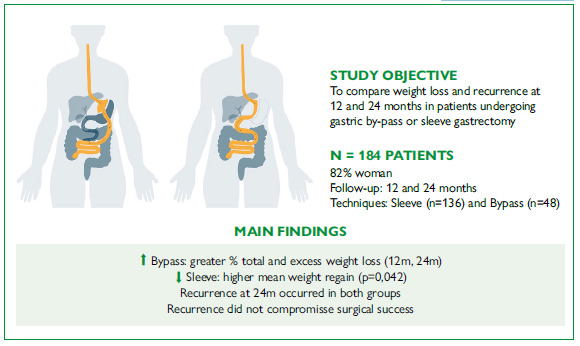

## RESUMO

**Racional:**

A cirurgia bariátrica é atualmente o padrão ouro no tratamento da obesidade, no entanto há recorrência de peso em diferentes técnicas empregadas.

**Objetivos:**

Comparar a evolução ponderal de pacientes, no 1º e 2º ano de pós-operatório, após a cirurgia bariátrica, submetidos às técnicas de bypass gástrico e gastrectomia vertical.

**Métodos:**

Estudo transversal, com adultos de ambos os sexos, acompanhados até 2 anos de cirurgia após bypass gástrico e gastrectomia vertical, analisando variáveis antropométricas associadas com características sociodemográficas, clínicas e de estilo de vida.

**Resultados:**

Foram avaliados 184 pacientes (gastrectomia vertical=136 e *bypass* gástrico=48), predominando o sexo feminino (82,1%). Ambos os grupos apresentaram boa adesão ao uso de polivitamínico, mas não à dieta e à atividade física. Após 1 e 2 anos de cirurgia, o percentual de perda de peso e percentual de perda do excesso de peso, foram maiores no bypass gástrico (p<0,001 e p=0,010 e p<0,001 e p<0,001 respectivamente). O ganho de peso médio foi de 2,37 Kg, sendo maior após a gastrectomia vertical (p=0,042), enquanto o percentual de recorrência ponderal não demostrou diferença entre as técnicas. A perda e recorrência de peso aos 2 anos, sofreram influência da dieta, em ambos os grupos. Houve maior percentual de perda de peso pós o bypass gástrico, após 1 e 2 anos de cirurgia e maior recorrência de peso após a gastrectomia vertical, sem interferir no sucesso cirúrgico da técnica.

**Conclusões:**

O bypass gástrico, demonstrou maior eficácia na perda ponderal, aos 12 e 24 meses. Houve recorrência de peso aos 24 meses em ambas as técnicas, sobretudo na gastrectomia vertical, sem configurar insucesso cirúrgico.

## INTRODUCTION

Bariatric surgery (BS) is considered the gold standard for the treatment of obesity that is difficult to control clinically and is recommended by the main guidelines due to its short- and long-term effectiveness^6^ ^,^ ^31^. BS is indicated based on the body mass index (BMI), whether or not associated with diseases induced and aggravated by obesity^6^. Among authorized surgical techniques, gastric bypass (GB) and gastric sleeve (GS) are the most widely performed throughout the world, with an increase in the indication of the latter^3^ ^,^ ^5^.

With GS, approximately 70% of the stomach is removed to restrict its capacity and, consequently, cause early satiety^3^ ^,^ ^21^. Hormonal changes also occur with the removal of portions of the stomach, especially the gastric fundus, which is where ghrelin is produced in greater quantities^30^. This orexigenic hormone is an important factor in reducing food intake^3^ ^,^ ^21^. Moreover, a reduction occurs in gastric acidity and vagal signaling is affected by the new gastric configuration, resulting in an increase in the stomach emptying rate and food transit to the duodenum, which leads to an increase in satiety hormones, such as GLP-1 and PYY, contributing to the effectiveness of the treatment of obesity and associated diseases^30^.

GB is a mixed technique with a restrictive component excluding approximately 70-80% of the stomach. The malabsorptive component involves diverting intestinal transit and promoting both a reduction in surface area for nutrient absorption and neuro-entero-hormonal changes, which reduce one’s appetite and increase satiety, leading to greater weight loss and better metabolic control and the control of chronic disease^2^ ^,^ ^11^ ^,^ ^17^ ^,^ ^30^.

These results of surgical interventions are satisfactory, with a significant reduction in excess body weight^24^. The criterion for diagnosing therapeutic success is the loss of at least 50% of excess weight as well as the maintenance of this loss in the long term^27^. However, surgical treatment can have unfavorable impacts such as weight recurrence (WR) due to various factors^16^ ^,^ ^24^. The recovery of more than 50% of the lost weight or the recovery of 20% or more of the lost weight combined with the return or development of comorbidities is considered indicative of surgical treatment failure^27^.

WR is mainly associated with unhealthy lifestyle and/or physiological and anatomical adaptations in the long term. Thus, studies whose authors seek to compare WR between the two main bariatric surgical techniques are important so that the indication of each technique can occur individually, thus ensuring the effectiveness of the surgical proposal and a better quality of life for patients^2^ ^,^ ^18^.

In the present study, we aimed to compare changes in weight in the first and second year following bariatric surgery in individuals submitted to the gastric bypass and gastric sleeve techniques.

## METHODS

A retrospective, cross-sectional case-series study was conducted involving patients from the nutrition/bariatric surgery outpatient clinic of the hospital affiliated with Universidade Federal de Pernambuco (UFPE). Patients of both sexes, 20-59 years of age, with BMI>35 kg/m² who underwent GS or GB between 2003 and 2019 were included.

Patients who underwent new abdominal surgeries, those with kidney disease, liver disease, AIDS, elevated edema, high degree of amputation, preoperative or postoperative plastic surgeries, those with illegible or incomplete records, those taking medications - such as chronic oral corticosteroids, steroids or growth hormone - in the pre- or postoperative period, and those on enteral and parenteral therapy in the postoperative period were excluded.

Data were collected on sociodemographic characteristics (age, sex, place of residence, and marital status), clinical characteristics (associated diseases, type of surgery, date of surgery, time since surgery, and complications), anthropometric characteristics (height, preoperative weight, late postoperative weight, BMI, ideal weight, excess weight [EW], excess weight loss [EWL], percentage of excess weight loss [%EWL], WR, and percentage of weight recurrence [%WR]), and lifestyle characteristics (adherence to the proposed dietary plan, physical activity, and use of multivitamins).

The data were descriptively analyzed using absolute and percentage frequencies for categorical variables, mean and standard deviation (mean±SD) values, and median with 25^th^ and 75^th^ percentiles (P25; P75). Either Pearson’s χ^2^ test or Fisher’s exact test were used, when applicable, for the comparison of categorical variables between the GS and GB groups. Either the Student’s t-test or Mann-Whitney U test were used for the comparison of continuous variables. Either the F-test (ANOVA) or Kruskal-Wallis test were used for the comparison of more than two categories. In the occurrence of significant differences using the F-test (ANOVA), Tukey’s test for multiple comparisons was applied. In the occurrence of significant differences using the Kruskal-Wallis test, Conover’s comparisons were applied. Normality and equality of variances were verified using the Shapiro-Wilk test and Levene’s F-test, respectively. The significance level was set at 5% (p<0.05). The data were entered onto an Excel spreadsheet and the Statistical Package for the Social Sciences (IBM SPSS) version 25 was used for statistical calculations.

This study was approved by the Human Research Ethics Committee of the Hospital affiliated with Universidade Federal de Pernambuco (UFPE) (certificate of approval number: 4.883.719, 08/04/2021).

## RESULTS

This study involved 184 adult patients (136 submitted to GB and 48 submitted to GS) out of a total of 738 patients followed up at the Nutrition/Bariatric Surgery Outpatient Clinic from 2003 to 2019. Patients who did not meet the eligibility criteria were excluded (414 with less than two years of nutritional follow-up; 74 who returned to outpatient follow-up only two years after surgery; one with high-grade edema; five pregnant women; two women on chronic high-dose corticosteroids; four patients underwent reconstructive surgeries, one was less than 20 years of age; 39 older people; eight who underwent new surgical approaches; three who underwent surgery at other services; one on enteral nutrition in the immediate postoperative period; and two who underwent revision surgery).

Women (82.1%), single/divorced or widowed individuals, those with less than eight years of education, and residents of the city of Recife predominated in the sample (Table 1). In Table 2, we describe the gastrointestinal variables that could interfere with weight loss as well as the rates of adherence to a healthy lifestyle.

**Table 1.** Sociodemographic and clinical characteristics of patients submitted to gastric sleeve and gastric bypass. Nutrition/Bariatric Surgery Outpatient Clinic.

Variable	Surgical technique			p-value
	Total Group	GB	GS	
	n (%)	n (%)	n (%)	
Age group (years)				
20 to 40	93 (50.5)	75 (55.1)	18 (37.5)	p*=0.036^#^
41 to 59	91 (49.5)	61 (44.9)	30 (62.5)	
TOTAL	184 (100.0)	136 (100.0)	48 (100.0)	
Sex				
Women	151 (82.1)	112 (82.4)	39 (81.3)	p*=0.864
Men	33 (17.9)	24 (17.6)	9 (18.8)	
TOTAL	184 (100.0)	136 (100.0)	48 (100.0)	
Marital status				
Single/Divorced/Widowed	92 (61.7)	69 (58.5)	23 (74.2)	p*=0.109
Married/Common-law marriage	57 (38.3)	49 (41.5)	8 (25.8)	
TOTAL	149 (100.0)	118 (100.0)	31 (100.0)	
Level of education (years)				
>8	19 (26.0)	14 (29.8)	5 (19.2)	p*=0.600
≤8	42 (57.5)	26 (55.3)	16 (61.5)	
No education	12 (16.4)	7 (14.9)	5 (19.2)	
TOTAL	73 (100.0)	47 (100.0)	26 (100.0)	
Place of residence				
Recife	82 (55.8)	63 (58.9)	19 (47.5)	p*=0.463
Metropolitan region	18 (12.2)	12 (11.2)	6 (15.0)	
Rural area	47 (32.0)	32 (29.9)	15 (37.5)	
TOTAL	147 (100.0)	107 (100.0)	40 (100.0)	
Comorbidities:				
Hypertension				
Yes	125 (67.9)	93 (68.4)	32 (66.7)	p*=0.827
No	59 (32.1)	43 (31.6)	16 (33.3)	
Diabetes mellitus				
Yes	50 (27.2)	37 (27.2)	13 (27.1)	p*=0.987
No	134 (72.8)	99 (72.8)	35 (72.9)	
Dyslipidemia				
Yes	9 (4.9)	4 (2.9)	5 (10.4)	p*=0.053
No	175 (95.1)	132 (97.1)	43 (89.6)	
Rheumatological diseases				
Yes	6 (3.3)	3 (2.2)	3 (6.3)	p**=0.184
No	178 (96.7)	133 (97.8)	45 (93.8)	
Respiratory diseases				
Yes	10 (5.4)	5 (3.7)	5 (10.4)	p**=0.130
No	174 (94.6)	131 (96.3)	43 (89.6)	
TOTAL	184 (100.0)	136 (100.0)	48 (100.0)	

GB: gastric bypass; GS: gastric sleeve; ^#^significant difference at 5.0% level; *Pearson’s χ^2^ test; **Fisher’s exact test.

**Table 2.** Clinical changes, adherence to dietary plan and physical activity in patients submitted to gastric sleeve and gastric bypass. Nutrition/Bariatric Surgery Outpatient Clinic.

Variable	Surgical technique			p-value
	Total Group	GB	GS	
	n (%)	n (%)	n (%)	
Signs and symptoms:				
Vomiting				
Yes	68 (37.0)	56 (41.2)	12 (25.0)	p*=0.046^#^
No	116 (63.0)	80 (58.8)	36 (75.0)	
Diarrhea				
Yes	17 (9.2)	16 (11.8)	1 (2.1)	p**=0.047^#^
No	167 (90.8)	120 (88.2)	47 (97.9)	
Constipation				
Yes	96 (52.2)	67 (49.3)	29 (60.4)	p*=0.184
No	88 (47.8)	69 (50.7)	19 (39.6)	
Dermatological				
Yes	127 (69.0)	98 (72.1)	29 (60.4)	p*=0.134
No	57 (31.0)	38 (27.9)	19 (39.6)	
Adherence to diet				
Yes	71 (38.6)	52 (38.2)	19 (39.6)	p*=0.913
Partially, with deviations	51 (27.7)	37 (27.2)	14 (29.2)	
No	62 (33.7)	47 (34.6)	15 (31.3)	
Use of multivitamins				
Yes	164 (89.1)	124 (91.2)	40 (83.3)	p*=0.133
No	20 (10.9)	12 (8.8)	8 (16.7)	
Practice of physical activity				
Yes	100 (54.3)	76 (55.9)	24 (50.0)	p*=0.482
No	84 (45.7)	60 (44.1)	24 (50.0)	
TOTAL	184 (100.0)	136 (100.0)	48 (100.0)	
Frequency of physical activity				
Daily	33 (33.0)	26 (34.2)	7 (29.2)	p*=0.420
≥3 times	57 (57.0)	41 (53.9)	16 (66.7)	
2 times	10 (10.0)	9 (11.8)	1 (4.2)	
TOTAL	100 (100.0)	76 (100.0)	24 (100.0)	

GB: gastric bypass; GS: gastric sleeve; ^#^significant difference at 5.0% level; ^(^Pearson’s χ^2^ test; **Fisher’s exact test .

In terms of weight loss, a significant difference was found between the two techniques at both one and two years after surgery (Table 3). The two groups had similar BMI values before and one year after surgery, whereas WR was greater in the GS group at the two-year follow-up.

**Table 3.** Anthropometric variables according to surgical technique and time since surgery. Nutrition/Bariatric Surgery Outpatient Clinic.

Variable	Surgical technique			p-value
	Total Group (n=184)	GB (n=136)	GS (n=48)	
	Mean±SD	Mean±SD	Mean±SD	
	Median (P25; P75)	Median (P25; P75)	Median (P25; P75)	
Ideal weight	64.27±8.28	64.07±7.97	64.83±9.20	p**=0.842
	62.95 (58.48; 68.61)	62.95 (58.29; 68.61)	63.74 (59.82; 69.03)	
Preoperative weight	119.13±24.74	120.15±25.21	116.22±23.36	p**=0.216
	114.95 (103.30; 132.80)	116.50 (103.85; 134.88)	110.50 (102.63; 128.83)	
Nadir weight	77.76±16.20	76.16±15.41	82.28±7.62	p**=0.049*
	76.50 (66.25; 86.98)	74.75 (64.70; 86.08)	79.00 (70.55; 90.30)	
Weight after 1 year	80.47±17.17	79.74±17.20	82.52±17.11	p**=0.307
	78.00 (68.20; 90.35)	76.55 (67.85; 90.40)	80.47 (70.15; 89.35)	
WL 1 year	38.66±14.27	40.41±13.97	33.70±14.10	p**=0.002*
	37.25 (29.80; 46.95)	39.05 (31.78; 47.98)	31.50 (24.00; 44.15)	
%WL 1 year	31.99±8.64	33.22±7.99	28.50±9.53	p**<0.001*
	32.65 (26.53; 38.10)	33.40 (28.90; 38.40)	28.40 (22.20; 33.10)	
%EWL 1 year	73.84±25.35	76.66±26.25	65.83±20.88	p**=0.010*
	71.56 (58.59; 86.82)	73.67 (61.42; 87.47)	64.55 (50.69; 81.91)	
Weight after 2 years	80.13±16.90	78.10±16.09	85.89±17.94	p**=0.011*
	78.85 (67.50; 91.20)	76.55 (66.00; 89.00)	82.25 (75.10; 94.03)	
WL 2 years	39.00±15.93	42.06±15.12	30.33±15.10	p**<0.001*
	38.15 (28.30; 48.10)	41.65 (33.40; 51.55)	28.50 (21.88; 37.30)	
%WL 2 years	32.09±10.04	34.41±8.75	25.52±10.63	p**<0.001*
	33.10 (26.40; 39.95)	35.55 (29.10; 40.83)	26.45 (19.18; 30.65)	
%EWL 2 years	73.35±25.69	78.40±24.21	59.04±24.56	p**<0.001*
	72.45 (57.28; 87.62)	77.07 (63.00; 90.11)	58.92 (42.33; 77.16)	
EW	54.86±20.48	56.09±21.62	51.39±16.54	p**= 0.109
	49.97 (41.75; 67.46)	53.63 (42.50; 68.58)	46.96 (39.59; 59.68)	
Preoperative BMI	46.17±7.58	46.75±8.23	44.51±4.99	p**=0.083
	44.81 (41.52; 50.19)	45.15 (41.67; 50.94)	43.91 (40.78; 48.68)	
BMI after 1 year	31.26±5.89	31.11±6.28	31.68±4.66	p**=0.248
	30.74 (27.35; 34.19)	30.46 (27.18; 33.80)	31.78 (27.81; 34.43)	
BMI after 2 years	31.12±5.70	30.45±5.69	33.03±5.32	p**=0.003*
	30.50 (26.90; 34.24)	29.90 (26.57; 33.56)	33.06 (28.83; 35.60)	
WR	2.37±3.75	1.94±3.09	3.61±5.02	p**=0.042*
	0.70 (0.00; 3.37)	0.50 (0.00; 3.17)	1.45 (0.00; 5.30)	
%WR 2 years	3.08±4.71	2.53±3.75	4.63±6.53	p**=0.062
	0.87 (0.00; 4.25)	0.72 (0.00; 4.20)	1.66 (0.00; 7.16)	

GB: gastric bypass; GS: gastric sleeve; WL: weight loss; %WL: percentage of weight loss; %EWL: percentage of excess weight loss; EW: excess weight; BMI: body mass index; WR: weight recurrence; %WR: percentage of weight recurrence; SD: standard deviation; BMI: body mass index; P25: 25th percentile; P75: 75th percentile; *Significant difference at 5.0% level; **Mann-Whitney U test.

Regarding adherence to a healthy lifestyle, %WL between 12 and 24 months exerted a significant influence on adherence to the diet (Table 4). Patients in the GB group lost more weight, especially those who followed the guidelines. In the analysis of weight loss influenced by physical activity (PA), both %WL in the two periods analyzed and weight gain were modified by adherence to PA (Table 5). WL associated with PA was higher in the GB group and the highest rate of WR was found among participants of the GS group without adherence to PA.

**Table 4.** Percentage of weight loss and gain at different postoperative assessments according to surgical technique and dietary plan. Nutrition/Bariatric Surgery Outpatient Clinic.

		n	Surgical technique		p-value
			GB (n=136)	GS (n=48)	
Variable	Response variable	GB/GS	Mean±SD	Mean±SD	
			Median (P25; P75)	Median (P25; P75)	
Diet:					
%WL 1 year	Follows	52/19	34.28±9.29	30.87±7.05	p^C^=0.049*
			34.55 (31.63; 40.10)	30.10 (25.60; 38.00)	
	Partial with deviations	37/14	32.28±6.40	30.35±9.73	p^C^=0.128
			31.70 (27.25; 37.90)	28.65 (24.85; 31.60)	
	Does not follow	47/15	32.80±7.57	23.77±10.87	p^D=^0.001*
			33.40 (27.50; 37.80)	22.20 (15.50; 28.80)	
	p-value		p^E^=0.167	p^E^=0.051	
%WL 2 years	Follows	52/19	36.27±9.40 ^(A)^	30.21±7.66 ^(A)^	p^C^=0.003*
			37.45 (32.55; 41.80)	27.80 (26.00; 33.60)	
	Partial with deviations	37/14	33.18±8.14 ^(B)^	28.38±8.53 ^(B)^	p^D=^0.069
			34.30 (27.70; 38.55)	27.20 (22.58; 32.58)	
	Does not follow	47/15	33.33±8.29 ^(B)^	16.93±10.92 ^(C)^	p^D^<0.001*
			34.40 (28.90; 39.20)	16.10 (7.90; 26.50)	
	p-value		P^E^=0.042*	p^F^<0.001*	
%WR 2 years	Follows	52/19	1.47±2.98 ^(A)^	0.78±1.20 ^(A)^	p^C^=0.750
			0.00 (0.00; 1.87)	0.00 (0.00; 1.36)	
	Partial with deviations	37/14	2.17±3.54 ^(A)^	2.62±3.32 ^(B)^	p^C^=0.323
			0.72 (0.00; 4.07)	1.14 (0.00; 3.82)	
	Does not follow	47/15	3.99±4.24 ^(B)^	11.39±7.63 ^(C)^	p^C^=0.001*
			3.38 (0.00; 6.00)	9.54 (4.34; 17.95)	
	p-value		p^E^=0.001*	p^E^<0.001*	
Multivitamin:					
%WL 1 year	Yes	124/40	33.12±8.09	29.16±9.26	p^C^=0.004*
			33.40 (28.83; 38.40)	28.65 (24.08; 33.10)	
	No	12/8	34.27±7.05	25.18±10.82	p^D=^0.035*
			32.70 (29.98; 39.08)	23.20 (16.80; 28.88)	
	p-value		p^C^=0.941	p^D^=0.285	
%WL 2 years	Yes	124/40	34.33±8.88	27.35±10.00	p^C^<0.001*
			35.80 (28.88; 40.60)	27.15 (21.50; 32.50)	
	No	12/8	35.25±7.64	16.41±9.35	p^D^<0.001*
			33.70 (29.43; 43.25)	17.60 (8.33; 25.63)	
	p-value		p^C^=0.918	p^D^=0.007*	
%WR 2 years	Yes	124/40	2.45±3.76	4.06±6.31	p^C^=0.297
			0.64 (0.00; 4.04)	1.12 (0.00; 4.98)	
	No	12/8	3.32±3.69	7.51±7.31	p^C^=0.134
			2.27 (0.04; 5.61)	6.27 (2.43; 9.21)	
	p-value		p^C^=0.215	p^C^=0.023*	

GB: gastric bypass; GS: gastric sleeve; %WL: percentage of weight loss; %WR: percentage of weight recurrence; SD: standard deviation; P25: 25th percentile; P75: 75th percentile; *Significant difference at 5.0% level; ^C^Mann-Whitney U test; ^D^Student’s t-test with equal variances; ^E^Kruskal-Wallis test with comparisons of referred test; ^F^F-test (ANOVA) with Tukey’s comparisons. Different letters in parentheses denote significant difference in adherence to diet by comparisons of referred test.

**Table 5.** Percentage of weight loss and gain at different postoperative assessments according to surgical technique and practice of physical activity. Nutrition/Bariatric Surgery Outpatient Clinic.

		N	Surgical technique		p-value
			GB (n=136)	GS (n=48)	
Variable	Response variable	GB/GS	Mean±SD	Mean±SD	
			Median (P25; P75)	Median (P25; P75)	
Physical activity:					
%WL 1 year	Yes	76/24	33.50±8.93	29.31±8.69	p^A^=0.029*
			33.40 (28.83; 38.70)	29.50 (21.93; 36.48)	
	No	60/24	32.87±6.66	27.68±10.43	p^B^=0.008*
			33.20 (29.40; 37.60)	27.95 (22.20; 30.08)	
	p-value		p^A^=0.465	p^B^=0.559	
%WL 2 years	Yes	76/24	34.20±9.14	27.78±8.80	p^A^=0.001*
			36.40 (28.80; 40.83)	27.30 (22.58; 31.98)	
	No	60/24	34.68±8.31	23.26±11.95	p^B^<0.001*
			34.70 (29.18; 40.90)	23.95 (16.55; 28.33)	
	p-value		p^A^=0.471	p^B^=0.142	
%WR 2 years	Yes	76/24	2.35±3.55	2.65±5.09	p^A^=0.670
			0.83 (0.00; 3.75)	0.74 (0.00; 2.46)	
	No	60/24	2.75±4.01	6.62±7.29	p^A^=0.004*
			0.47 (0.00; 4.47)	3.62 (1.08; 10.39)	
	p-value		p^A^=0.855	p^A^=0.008*	

GB: gastric bypass; GS: gastric sleeve; %WL: percentage of weight loss; %WR: percentage of weight recurrence; SD: standard deviation; P25: 25th percentile; P75: 75th percentile; *Significant difference at 5.0% level; ^A^Mann-Whitney test; ^B^Student’s t-test with equal variances.

## DISCUSSION

Bariatric surgery offers excellence in the treatment of severe obesity, providing effective, long-lasting control of the condition and associated metabolic diseases^6^ ^,^ ^9^ ^,^ ^31^. However, the biggest obstacle currently encountered is weight recurrence (WR), which remains a challenge despite decades of surgical improvements. The exact mechanisms of WR are not yet fully understood, but it is believed to involve the sum of several factors, particularly behavioral and physiological aspects^2^ ^,^ ^24^ ^,^ ^35^.

The sociodemographic characteristics of the groups were similar in terms of age range, as described by other authors in studies conducted in Northeast Brazil^25^ ^,^ ^32^. There is a consensus in the literature that women predominate at all bariatric surgery services, as occurred in the present study^1^ ^,^ ^7^ ^,^ ^8^ ^,^ ^13^ ^,^ ^14^ ^,^ ^20^ ^,^ ^28^. The patients submitted to GB had a higher occurrence of gastrointestinal symptoms, which is in agreement with data described in the literature^15^.

In this study, we compared the effectiveness of two surgical techniques, analyzing WL, %WL, %EWL, WR, and %WR one and two years postoperatively in patients submitted to surgery in the period from 2003 to 2019. Preoperative anthropometric characteristics were similar between the groups, with no significant differences in weight, ideal weight, excess weight, or BMI, which makes this study important in comparing changes in weight between the techniques. Regarding %WL, authors of studies conducted in New Zealand and Norway found greater weight loss at one year in individuals submitted to GB compared to those submitted to GS (29-32.2% versus 23-27.1%, respectively), which is similar to our findings^12^ ^,^ ^17^.

A French study whose authors investigated %EWL between these techniques found no significant differences in the first and second year after bariatric surgery. However, the GB group had a higher %EWL than the GS group from the third year onwards (83 and 66.3%, respectively)^13^. Similarly, Seetharamaiah et al. found no significant difference in the first postoperative year (GB=66.8 vs. GS=63.9, with p>0.05)^29^. These results differ from those of the New Zealand study, which demonstrated a higher %EWL in the GB group in this period (GB=84.2 vs. GS=70.2%). This also differs from the results of a systematic review of seven studies involving a total of 3,862 patients, in which the GB technique promoted a higher %EWL, with values ​​ranging from 37.1 to 92.2% vs. 31.4 to 71.4% with the GS technique (p=0.005), confirming the findings of the present investigation^17^ ^,^ ^34^. Authors of a study conducted in the city of Ribeirão Preto, Brazil, investigating changes in weight in patients submitted to GB up to 10 years after surgery, found weight reduction (5.5%) up to the second year and the maintenance of this weight loss up to the following three years^19^.

Weight loss (WL) was significantly greater in the GB group. However, the literature commonly assesses the change in weight after bariatric surgery considering %WL and %EWL. The same occurs with BMI, as values are often analyzed as the loss of excess BMI. Authors of two Swiss studies compared the loss of excess BMI in GB and GS. In the first, researchers found that both techniques were statistically similar in the first and second years (76.6-77.7% vs. 72.3-74.7% in GB and GS, respectively)^18^, whereas in the second the authors found a greater loss of excess BMI in the GB group in 17 months (76.6 vs. 64.4%, p<0.046)^28^. Similarly, we found a lower BMI in the GB group two years after surgery.

According to the Brazilian Society of Bariatric and Metabolic Surgery, after the stabilization of weight loss, it is tolerable for patients to regain between 10 and 15% of the lowest weight achieved after surgery, which is denominated the *nadir* ^27^. The definition of WR varies widely in the literature. Abdallah et al.^1^, in a systematic review, found a 5.7% WR after two years in patients submitted to the GS technique, which is much higher than the rate found in the present study (median of 1.66% in GS). The authors defined WR as recovery greater than 10 kg from the nadir^1^ ^,^ ^35^. Authors of a study conducted in South Africa defined WR as the recovery of 5% or more from the nadir and found that 10.1% of the patients submitted to GS experienced WR at two years^8^. Conversely, authors of a study conducted in the United States, in which 95% of the patients were submitted to GS, defined WR as the recovery of 20% or more from the nadir and detected a 34.7% WR rate at the end of three years^7^.

In the present study, WR was greater in the GS group in terms of absolute weight values. However, the same did not occur in the statistical comparison of %WR between the two techniques. Therefore, it could be inferred that the GS group, despite having greater WR values, did not experience surgical treatment failure, as the %WR was below the reference value of the Brazilian Society of Bariatric and Metabolic Surgery^27^.

According to Tabesh et al.^33^, good adherence to the diet prevents the occurrence of gastrointestinal complications, malnutrition, and WR and is therefore an essential factor for adequate weight loss and maintenance in the short and long terms. Evidence indicates that 60% of patients submitted to bariatric surgery are lost to nutritional follow-up^2^. In a randomized clinical trial, Nijamkin et al.^18^ demonstrated that patients submitted to GB who received sessions of nutritional education for six weeks obtained a significantly greater %EWL after one year compared to the group given the usual treatment.

Authors of a meta-analysis of five studies involving a total of 994 patients found that snacking was identified in 16.6 to 46.6% of the sample and the prevalence of WR was 47%^20^. This eating behavior can cause dilation of the gastric pouch^2^. Researchers demonstrated that the gastric volume increased from 120 mL to 524 mL in patients five years after GS surgery and WR, significantly reducing the restrictive potential of surgery^4^. In the present investigation, low adherence to the diet proposed by the nutritionist had a negative impact in both groups, with a greater impact in the GS group.

Food quality and the distribution of macronutrients in meals are deemed fundamental for the effective maintenance of weight lost^2^. WR is associated with excessive food intake, with a predominance of high-calorie snacks and foods outside the meal plan^2^. Analyzing women with WR greater than 5% of the nadir submitted to supplementation with whey protein for 16 weeks, Gomes et al.^10^ found an average weight loss of 1.86 kg and a reduction in body fat with the preservation of muscle, whereas the non-supplemented group had an average gain of 0.42 kg in the period. In the present investigation, non-adherence to vitamin and mineral supplementation exerted an influence on %WL at the two-year follow-up in the GS group. The lack of studies analyzing this variable impedes the comparison of this finding.

While physical activity (PA) is considered an important measure for improving WL efficiency and preventing WR after bariatric surgery, only 10 to 24% of patients adhere to the minimum weekly exercise time established in the guidelines^2^ ^,^ ^22^. A meta-analysis investigating the influence of PA found lower WL in the sedentary group and an additional loss of 1.9 kg in the active group^22^. PA has been positively associated with better outcomes, particularly aerobic-resistance exercises, which result in high energy expenditure^2^. Moreover, strengthening exercises promote muscle synthesis, which, in turn, increases energy expenditure at rest^23^. In the present study, we verified a positive association between greater weight loss and PA adherence in both groups and greater %WR was found among sedentary patients in the GS group. However, there is a lack of studies comparing this difference between surgical techniques^22^.

Other factors not investigated in this study are related to the effectiveness of WL and the control of WR in the medium and long terms after bariatric surgery^2^. Hormonal changes (increase in ghrelin and leptin and reduction in incretins), which can occur over time, and mental health issues (depression or eating disorders) are associated with worse outcomes^2^ ^,^ ^26^. Therefore, it can be inferred that bariatric surgery alone is not sufficient for the control of obesity and associated diseases. A better understanding of hormonal, psychological, behavioral, and surgical mechanisms is necessary to explain how the combination of these factors contributes to weight loss and WR with each technique individually^2^.

## CONCLUSIONS

Surgical technique exerted an influence on %WL and %EWL at 12 and 24 months. Gastric bypass was better for achieving the surgical objective in these two periods. Weight recurrence occurred only at 24 months in both groups and was greater among the patients submitted to the gastric sleeve technique, but without constituting surgical failure. These results are relevant and studies analyzing long-term weight recurrence are needed.

## Data Availability

The information regarding the investigation, methodology and data analysis of the article is archived under the responsibility of the authors.
